# Sex-Specific Associations of Famine Exposure in Early Life with Body Shape Indices Across Two Generations in China

**DOI:** 10.3390/nu18152544

**Published:** 2026-08-04

**Authors:** Weiyuan Yao, Leah Li, Yongfu Yu, Wanghong Xu

**Affiliations:** 1 Department of Chronic Disease Prevention and Control, Zhejiang Provincial Center for Disease Control and Prevention, 3399 Binsheng Road, Hangzhou 310051, China; ywyao@cdc.zj.cn; 2 Department of Epidemiology, NHC Key Laboratory for Health Technology Assessment, Fudan University School of Public Health, 138 Yi Xue Yuan Road, Shanghai 200032, China; 3 Population, Policy and Practice Research and Teaching Department, University College London Great Ormond Street Institute of Child Health, London WC1N 1EH, UK; leah.li@ucl.ac.uk; 4 Department of Biostatistics, NHC Key Laboratory for Health Technology Assessment, Key Laboratory of Public Health Safety of Ministry of Education, Fudan University, Shanghai 200032, China; yu@fudan.edu.cn; 5 Yiwu Research Institute of Fudan University, Building V of Zhongfu Square, Yiwu 322000, China

**Keywords:** famine exposure, early life, body shape, intergenerational association

## Abstract

**Background/Objectives**: Early-life undernutrition may exert lifelong and intergenerational effects on height and weight, but its impact on body shape remains unclear. Using the Great Chinese Famine (1959–1961) as a natural experiment, this study explored the associations between early-life undernutrition and body shape indices across two generations. **Methods**: We analyzed nine waves of longitudinal data from the China Health and Nutrition Survey (1991–2015), including 5156 participants (F1) born in 1955–1966 and their 2383 biological offspring (F2). F1 famine exposure was classified by birth year; F2 exposure was defined by parental status. Anthropometric indices including body adiposity index (BAI), body roundness index (BRI), waist-to-height ratio (WHtR), conicity index (COI), and a body shape index (ABSI) were derived from repeated measurements. Age-specific fitted curves of these indices were compared across exposure groups using mixed-effects models with fractional polynomials of age. **Results**: Fetal famine exposure was modestly associated with lower COI and ABSI in adulthood, particularly among men. Childhood famine exposure was significantly linked to lower BAI (−0.40), BRI (−0.26), WHtR (−0.0140), COI (−0.0188), and ABSI (−0.0008), with stronger associations in men (all P for sex interaction <0.05). Intergenerationally, maternal childhood exposure was associated with higher offspring BAI, whereas paternal childhood exposure was associated with lower BAI, BRI, WHtR, COI, and ABSI. **Conclusions**: Famine exposure showed more pronounced associations with body shape in men, and maternal and paternal exposures displayed different intergenerational patterns. These exploratory findings suggest the possibility that early-life undernutrition may relate to long-term phenotypes in a sex-specific manner, though this requires independent replication.

## 1. Introduction

The Developmental Origins of Health and Disease (DOHaD) hypothesis posits that adverse early-life exposures, including nutritional deficiencies during gestation, early childhood, and even early adulthood, may predispose individuals to cardio-metabolic diseases later in life [1,2]. Extensive evidence links early-life undernutrition to childhood growth and health outcomes, which may subsequently elevate the risk of cardio-metabolic disorders in adulthood [3]. Beyond these lifelong effects [2], animal experiments indicate that the consequences of early-life nutrition could extend to subsequent generations through epigenetic mechanisms [4,5]. In mice, for example, caloric restriction during late gestation (F0) resulted in low birth weight, obesity, and impaired glucose intolerance in the next two generations (F1 and F2) [6].

In recent decades, several studies have used historical famines as natural experiments to investigate the intergenerational impacts of early-life adverse exposures on human offspring [7,8,9,10,11]. For instance, the Dutch famine study reported increased adiposity in male offspring of fathers who experienced prenatal undernutrition [7]. In northern Sweden, offspring of grandfathers exposed to famine during pre-puberty showed lower all-cause mortality than those with unexposed grandfathers [8]. Our recent studies have shown that early-life exposure to the Chinese Great Famine was associated with reduced body height across two consecutive generations [9] and with an elevated obesity risk in offspring of exposed mothers, as assessed by body mass index (BMI) and waist circumference [10,11]. These findings suggest sex-specific and intergenerational associations between early-life nutritional deficiency and obesity-related phenotypes.

However, traditional indices, such as BMI and waist circumference, are insufficient for accurately reflecting whole-body fat distribution or distinguishing muscle from fat mass [12]. Similarly, although body height is associated with cardio-metabolic disease risk [3], it is not an optimal indicator of individual health status [13]. Recently, several novel body shape indices, including the waist-to-height ratio (WHtR), body adiposity index (BAI), body roundness index (BRI), conicity index (COI), and a body shape index (ABSI), have been developed as complementary anthropometric proxies for body composition, visceral adiposity, and central fat accumulation, all of which are closely related to metabolic dysfunction [14,15,16,17,18]. These indices integrate multiple anthropometric measurements, such as weight, height, waist circumference, and hip circumference, to more accurately assess body shape and composition [14,15,16,17,18]. Compared with BMI, these indices have demonstrated stronger associations with metabolic syndrome and cardiovascular disease risk [18]. Specifically, BRI has outperformed other indices in predicting metabolic syndrome risk in both sexes [19], whereas ABSI has shown a closer association with all-cause mortality [18]. Additionally, WHtR has been associated with hypertension and cardiovascular disease risk [20]. Despite well-documented links between these indices and later disease risk, and between early-life malnutrition (both in utero and during infancy) and adult health status, few prospective studies have examined the potential influence of early-life malnutrition on these body shape indices across generations.

In this study, we used longitudinal data from the China Health and Nutrition Survey (CHNS) to examine the associations between early-life exposure to the Chinese Great Famine, a proxy for early-life malnutrition, and several novel body shape indices (BAI, BRI, WHtR, COI, and ABSI) in individuals born during 1955–1966 and in their offspring. Given that the offspring of famine-exposed individuals have not yet reached the peak age for cardio-metabolic diseases, these associations may provide insights into the long-term effects of early-life undernutrition on disease risk.

## 2. Materials and Methods

### 2.1. Study Design and Participants

The CHNS is an ongoing, household-based open cohort study initiated in 1989, with nine survey waves conducted in 1991, 1993, 1997, 2000, 2004, 2006, 2009, 2011, and 2015. Participants were recruited from 12 provinces using multi-stage cluster random sampling of households to ensure national representativeness [21,22].

**Figure 1:**
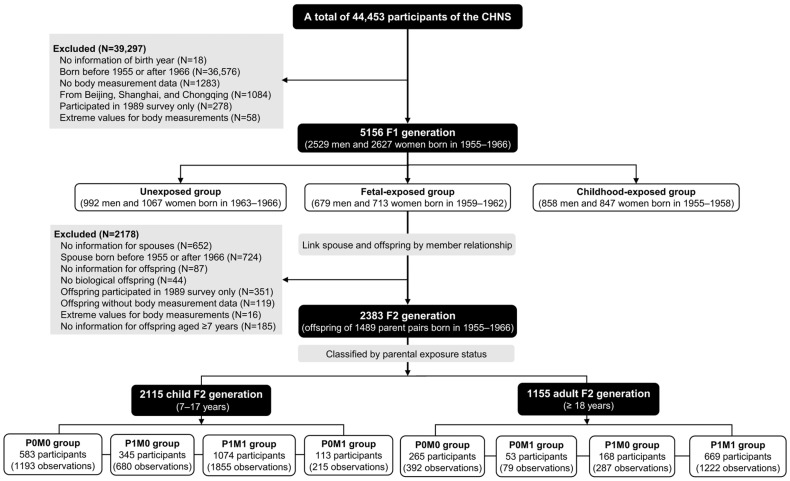
Flow chart for the selection of study participants. P0M0, neither parent exposed to famine; P1M0, paternal exposure only; P0M1, maternal exposure only; P1M1, both parents exposed to famine.

The Great Chinese Famine occurred during 1959–1961. To minimize between-group age differences and secular trends in adiposity, we restricted the F1 generation to individuals born between 1955 and 1966, consistent with previous studies [23]. Eligible F1 participants were identified after excluding those who: (1) were born before 1955 or after 1966; (2) lacked birth date or body measurement data; (3) participated only in the 1989 survey (which used different protocols and instruments from subsequent waves); and (4) resided in Beijing, Shanghai, or Chongqing (cities not included in the sampling framework before 2009). F2 participants were identified as the biological offspring of eligible F1 participants through household IDs and kinship questionnaires. Individuals with extreme anthropometric values were further excluded. The final sample comprised 5156 F1 and 2383 F2 participants with at least one valid anthropometric measurement (Figure 1). Overall, 3909 F1 (75.8%) and 1757 F2 (73.7%) participants had ≥2 valid anthropometric measurements, with comparable proportions across exposure groups (73.2–78.2% and 71.7–77.5%, respectively).

The CHNS protocols, instruments, and informed consent procedures were approved by the Institutional Review Committees of the University of North Carolina at Chapel Hill, the National Institute of Nutrition and Health, and the Chinese Centre for Disease Control and Prevention. CHNS data and study materials are publicly available at https://www.cpc.unc.edu/projects/china (accessed on 1 February 2025).

### 2.2. Definition of Famine Exposure

The Great Famine (1959–1961) affected nearly all regions of mainland China [24]. However, the onset, duration, and severity varied across areas and were not uniformly documented. Previous reports indicate that overall mortality in the nine provinces covered by the CHNS rose from 1959, peaked in 1960, and returned to pre-famine levels (comparable to 1956–1958) by 1962 [25]. Accordingly, F1 famine exposure was defined by birth year: unexposed (1963–1966), fetal-exposed (1959–1962), and childhood-exposed (1955–1958) [9,10,11]. For F2 participants, grouping was based on parental exposure status. Offspring of parents both born during 1955–1966 were classified into four groups: (1) neither parent exposed (P0M0); (2) only mother exposed (P0M1); (3) only father exposed (P1M0); and (4) both parents exposed (P1M1).

### 2.3. Body Shape Indices

At each survey wave, trained staff measured height, waist circumference, and hip circumference following standardized protocols [21,22,26]. Height was measured without shoes to the nearest 0.1 cm using a portable stadiometer; weight was measured without shoes and in light clothing to the nearest 0.1 kg using a calibrated beam scale; waist circumference was measured at the midpoint between the inferior rib margin and the iliac crest at the end of exhalation. Participants removed bulky clothing and shoes before measurement. Body shape indices were calculated using the following formulas:

1. $BAI\;=\;\frac{{hip\;circumference}_{\left(cm\right)}}{{{height}^{1.5}}_{\left(m\right)}}-18$ [17]
2. $BRI\;=\;364.2-365.5*\sqrt{1-\frac{{\left({waist\;circumference}_{\left(m\right)}/2\pi \right)}^{2}}{{\left(0.5*{height}_{\left(m\right)}\right)}^{2}}}$ [16]
3. WHtR = waist circumference(cm)/height(cm) [27]
4. $COI\;=\;{waist\;circumference}_{\left(m\right)}/\left(0.109\;*\;\sqrt{\frac{{body\;weight}_{\left(kg\right)}}{{height}_{\left(m\right)}}}\right)$ [15]
5. $ABSI\;=\;{waist\;circumference}_{\left(m\right)}/\left({BMI^{\frac{2}{3}}}_{\left(kg/m^{2}\right)}\;*\;{{height}^{\frac{1}{2}}}_{\left(m\right)}\right)$ [14]

$ABSI\;(foradolescentaged\;7\;\sim \;18\;years)\;=\;{waist\;circumference}_{\left(m\right)}/\left({BMI^{0.45}}_{\left(kg/m^{2}\right)}\;*\;{{height}^{0.55}}_{\left(m\right)}\right)$ [28].

### 2.4. Covariates

Covariates included age at survey, sex, educational level, physical activity, energy intake, residential area, famine severity, alcohol use, and smoking status. For F1, education was dichotomized as low (junior high or below) or high (senior high or above). For F2, parental education was classified as lower-level (both parents junior high or below) or higher-level (at least one parent senior high or above). Physical activity was assessed using a self-constructed questionnaire [21] and classified as light, moderate, or heavy for adults [9] or as low or high for participants aged 7–18 years. Energy intake was estimated from three consecutive 24 h diet recalls and expressed as kcal/day [29]. Residential area was classified as rural or urban. Famine severity was defined by excess mortality in each province during 1959–1961. Henan, Hunan, Guangxi, and Guizhou were classified as more severely affected, and Liaoning, Heilongjiang, Jiangsu, Shandong, and Hubei as less severely affected [30]. Alcohol and tobacco were coded as yes or no for F1 and adult F2 participants [21].

### 2.5. Statistical Analysis

Participant characteristics were described using mean (standard deviation) for continuous variables and frequency (percentage) for categorical variables. Group comparisons across famine exposure categories used analysis of variance for approximately normally distributed continuous variables, Kruskal–Wallis tests for skewed continuous variables, and chi-square tests for categorical variables.

Mixed-effects models were used to examine associations between famine exposure and each body shape index, with repeated measurements (level 1) nested within individuals (level 2) and individuals nested within households (level 3) to account for sibling correlation [31]. Fractional polynomial functions were employed to model nonlinear relationships between the indices and age [32]. The best-fitting fractional polynomials (age, age^2^, and age^3^ for F1; age and age^2^ for adult F2; and age and age^2^ × log_10_(age) for child F2) were selected using the Akaike information criterion. Regression coefficients (β) and 95% confidence intervals (95%CI) were estimated to quantify differences in body shape indices between exposed and unexposed groups.

Among three socioeconomic indicators (education, occupation, and income), F1 educational attainment was relatively stable after childbirth and was therefore used as a childhood socioeconomic indicator for F2 and a proxy of socioeconomic status for F1 [9]. A directed acyclic graph (DAG) was used to inform covariate selection (Figure S1). Age and sex were included because body shape indices vary across the life course and by sex, and because body composition and famine vulnerability differ by sex. Urban/rural residence, education (or parental education for F2), and province-level famine severity were included as covariates to capture socioeconomic and contextual factors that may affect both famine exposure and anthropometric outcomes. Behavioral factors (alcohol use, smoking, energy intake, and physical activity) were considered potential post-exposure variables and excluded from final models, as additional adjustment did not materially change the estimates. Final models were adjusted for sex, age, residential area, famine severity, and F1 education (parental education for F2). Given minimal difference between 2- and 3-level models, 2-level results were reported. There were no missing values for famine exposure or other covariates in the final analyses.

Previous studies reported significant sex- and age-heterogeneity in intergenerational associations between famine exposure and anthropometrics [33,34]. We therefore conducted subgroup analyses stratified by five-year age groups for F1 and by ages 11, 18, and 25 years for F2 to evaluate potential modifying effects of age. We also tested sex-heterogeneous effects by examining interactions between sex and famine exposure in F1 and between offspring sex and parental exposure status. Because our previous study reported a significant interaction between maternal and paternal exposure [11], we tested the interaction in the current study. The statistical significance of effect modification was evaluated by including multiplicative interaction terms (i.e., age × famine) in the mixed-effects models with adjustment for covariates.

We performed three sensitivity analyses to test robustness: (1) restricting analyses to rural participants; (2) excluding F1 individuals born in 1958 or 1963 and their offspring to avoid potential exposure misclassification; and (3) combining the unexposed (1963–1966) and childhood-exposed (1955–1958) groups as an age/birth-balanced control for the fetal-exposed group (1959–1962) to minimize cohort and age effects [23].

All analyses were performed using SAS version 9.4 (SAS Institute Inc., Cary, NC, USA) and R version 4.3 (R Foundation for Statistical Computing, Vienna, Austria).

## 3. Results

### 3.1. Demographics of F1 and F2 Participants

Among F1, famine-exposed individuals were older and less physically active than the unexposed (Table 1). Fetal-exposed participants were less likely to reside in severely affected areas and more likely to have attained higher education than the unexposed or childhood-exposed. Famine-exposed men were more likely to smoke and drink, while childhood-exposed women had higher smoking rates. No significant differences were observed in residential areas or energy intake.

For F2, offspring of exposed parents were older, born earlier, had better-educated parents, and engaged in more heavy physical activity than the P0M0 group; they were also more likely to reside in rural areas or severely affected regions (Table S1).

### 3.2. Early-Life Famine Exposure and Body Shape Indices in F1

**Figure 2:**
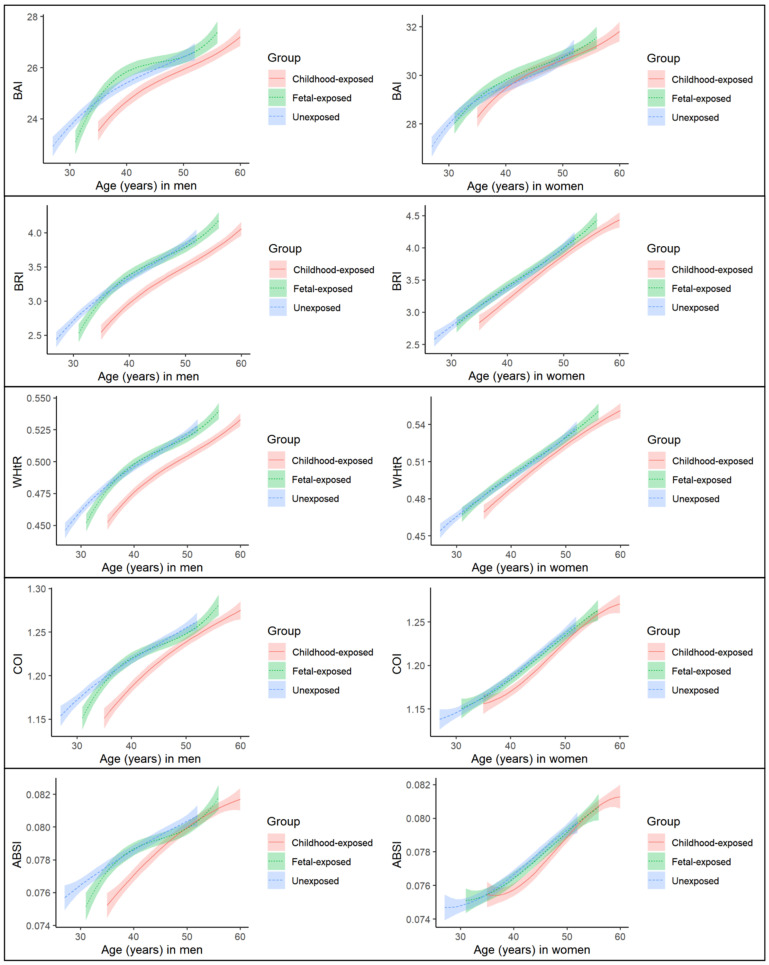
Age-specific fitted curves of body shape indices for F1 men (**left**) and women (**right**) by early-life famine exposure. Abbreviations: BAI, body adiposity index; BRI, body roundness index; WHtR, waist-to-height ratio; COI, conicity index; ABSI, a body shape index. Age-specific fitted curves fitted using linear mixed models with fractional polynomials for age (years) as fixed effects and participant-specific random intercepts.

All body shape indices increased monotonically with age among F1 participants, but childhood-exposed individuals had significantly lower mean levels (*p*-values < 0.05) (Figure 2). In men, age-specific fitted curves for BAI, COI, and ABSI differed significantly by exposure status (*p*-values for interaction with age < 0.05).

**Figure 3:**
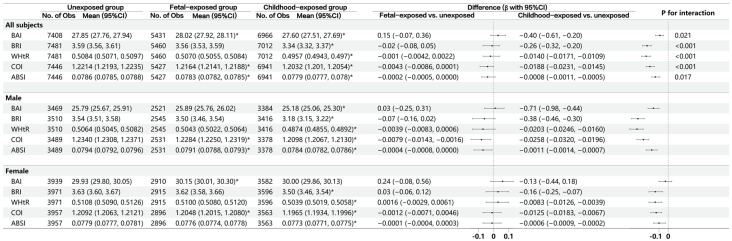
Associations of early-life famine exposure with body shape indices later in life among the F1 generation. Abbreviations: BAI, body adiposity index; BRI, body roundness index; WHtR, waist-to-height ratio; COI, conicity index; ABSI, a body shape index; CI, confidence interval. No. of Obs: number of available observations for each body shape index of the eligible participants. Mean (95%CI) estimated using linear regression models adjusting for age at survey and sex. * *p* < 0.05 compared with the unexposed group, adjusting for age and sex. The β with 95%CI represents the difference in each body shape index between the exposed and unexposed groups, estimated using linear mixed effects models with fractional polynomials for age and adjusted for sex, residential areas (urban or rural), educational level (high or low), and severity of famine (less or more). *p* for interaction between famine exposure and sex. The values of WHtR, COI, and ABSI are presented with four decimal places to reflect the small differences between groups. Results are presented in the forest plots derived from the standardized values (z-scores) for each index.

Age- and sex-adjusted means of all five indices were significantly lower in childhood-exposed than unexposed participants (*p*-values < 0.05) (Figure 3). Specifically, fetal-exposed men had lower mean COI and ABSI, whereas fetal-exposed women had higher mean BAI.

After full adjustment, childhood-exposed individuals showed significantly lower BAI, BRI, WHtR, COI, and ABSI than the unexposed. These associations were stronger in men (all *p*-values for sex interaction < 0.05). Conversely, fetal-exposed men showed only marginally lower COI and ABSI, with no other significant differences.

To account for age variation, indices measured at different ages were compared (Figure S2). Childhood-exposed men had lower indices at nearly all ages, with differences narrowing after age 40. Fetal-exposed men showed lower indices at ages 30–34, but higher BAI at ages 40–44 (*p*-values < 0.05); a similar but weaker pattern was seen in women. Significant exposure-by-age interactions were observed for all five indices (all *p*-values < 0.05).

### 3.3. Famine Exposure and Body Shape Indices in Child Offspring

Age-specific fitted curves overlapped substantially regardless of parental famine exposure. Most indices were comparable among female offspring across exposure groups (Table S2). However, male offspring of mothers exposed during the fetal or childhood periods had lower mean BAI (*p*-values < 0.05).

**Figure 4:**
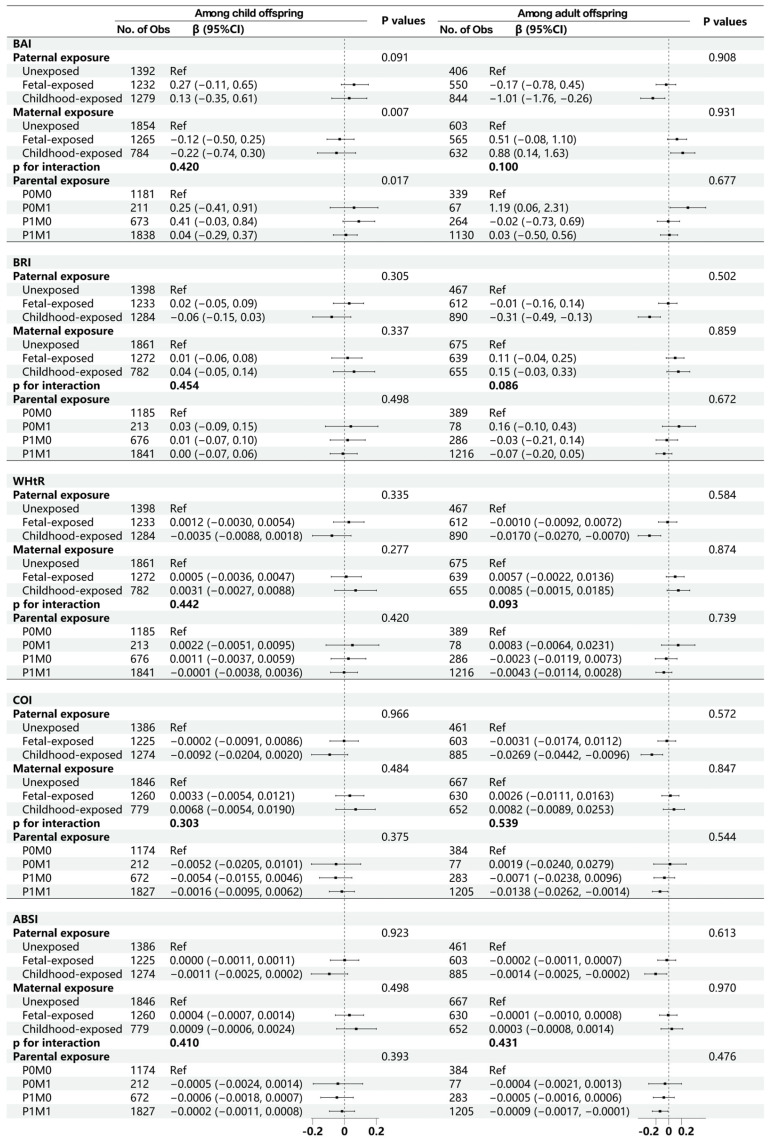
Associations of parental early-life famine exposure with body shape indices in offspring. Abbreviations: BAI, body adiposity index; BRI, body roundness index; WHtR, waist-to-height ratio; COI, conicity index; ABSI, a body shape index (left for adolescents); CI, confidence interval; No. of Obs, available observations for each body shape index of the eligible participants; P0M0, neither parent exposed to famine; P1M0, paternal exposure only; P0M1, maternal exposure only; P1M1, both parents exposed to famine. The β with 95%CI represents the difference in each body shape index between the exposed and unexposed groups, estimated using linear mixed effects models with fractional polynomials for age and adjusted for sex, residential areas (urban or rural), educational level (high or low), and severity of famine (less or more). *p* values for the interactions between paternal, maternal, and parental famine exposure and sex. P for interaction between paternal and maternal famine exposure. The values of WHtR, COI, and ABSI are presented with four decimal places to reflect the small differences between groups. Results are presented in the forest plots derived from the standardized values (z-scores) for each index.

After full adjustment, no significant associations were observed between parental famine exposure and offspring childhood indices (Figure 4). Sex-stratified analysis revealed that male offspring of fetal-exposed mothers had lower BAI (*p*-values < 0.05 for sex-heterogeneity; Table S3). No significant maternal-paternal exposure interaction was observed (all *p*-values > 0.05).

### 3.4. Famine Exposure and Body Shape Indices in Adult Offspring

Age-specific fitted curves did not differ significantly by parental exposure in adult offspring. However, offspring of childhood-exposed fathers had significantly lower BRI, WHtR, and COI than those with unexposed fathers (Table S4).

After adjustment, maternal childhood exposure was associated with higher offspring BAI, whereas paternal childhood exposure was associated with lower BAI, BRI, WHtR, COI, and ABSI (Figure 4). No significant heterogeneity was detected by offspring sex or by maternal versus paternal exposure.

Further analysis showed that the P0M1 group had higher BAI, whereas the P1M1 group had lower COI and ABSI relative to P0M0. Parental exposure timing yielded similar intergenerational patterns (Figure S3).

### 3.5. Famine Exposure and Body Shape Indices in Offspring by Age Periods

No significant differences were observed between paternal fetal-exposed and unexposed groups across age periods (Figure S4). However, differences between paternal childhood-exposed and unexposed groups widened with age; offspring of childhood-exposed fathers aged ≥25 years showed lower BAI, WHtR, and COI. By contrast, indices in offspring of maternal fetal- and childhood-exposed groups progressively exceeded those of the unexposed group with increasing age. Overall, the P0M1 group showed higher BAI, BRI, and WHtR than P0M0 at ages ≥25 years, whereas the P1M1 group showed lower COI and ABSI at ages 18–24 years. Significant parental exposure-by-age interactions were observed for all five indices (all *p*-values < 0.05).

### 3.6. Sensitivity Analyses

Results remained largely unchanged when analyses were restricted to rural participants or excluded individuals born in 1958 or 1963 (Figure S5). Exclusion of urban F2 participants or offspring of parents born in 1958 or 1963 also yielded similar findings (Figures S6 and S7). However, compared with the age/birth-balanced control group, fetal-exposed participants and offspring of fetal-exposed parents showed higher indices (Figures S6–S8).

## 4. Discussion

Using longitudinal data from two generations, we examined associations between early-life famine exposure and multiple body shape indices in exposed individuals and their offspring. We found that childhood famine exposure was negatively associated with later-life body shape indices, particularly in men; that maternal childhood exposure was linked to higher offspring BAI, whereas paternal childhood exposure was associated with lower BAI, BRI, WHtR, COI, and ABSI; and that intergenerational associations were more pronounced for BAI, BRI, and WHtR than for COI and ABSI.

Previous studies generally report that early-life famine exposure increases the risks of obesity and cardio-metabolic disease relative to the unexposed group [23,35,36]. However, these estimates are highly sensitive to control group selection [23]. For instance, one cross-sectional study found higher BMI in famine-exposed individuals, but the exposed group was 7–15 years older than controls, confounding age with exposure [35]. Another study using the China Health and Retirement Longitudinal Study (CHARLS) defined fetal-exposed (born in 1959–1961) and control (born in 1955–1957, i.e., childhood-exposed) groups from different survey waves, thereby failing to disentangle fetal from childhood effects [36]. In this study, sensitivity analyses using an age/birth-balanced control group (including both unexposed and childhood-exposed participants) also showed positive associations between fetal exposure and the indices. Although this improved birth-year balance, it could not distinguish the potential influence of fetal exposure from that of childhood exposure.

By contrast, our longitudinal design used an unexposed reference group and addressed age-confounding through age-specific fitted curves and stratified analysis. Strikingly, we found that childhood famine exposure was associated with lower body shape indices. This apparent contradiction aligns with evidence linking low birth weight to reduced adult obesity risk [37], and with Dutch famine data showing lower obesity risk at age 19 among those exposed during late gestation and early infancy [38]. Nutritional deprivation during critical developmental periods may alter adipose-tissue cellularity [38]. Our childhood-exposed cohort (born 1955–1958) likely experienced delayed catch-up growth due to prolonged post-famine poverty until the 1978 economic reforms. This delay may have prevented the adiposity rebound typically observed after nutritional rehabilitation [39,40].

Consistent with this interpretation, animal studies indicate that continued postnatal dietary restriction can yield normal adult body weight and fat levels [40], and that such restriction may improve health and longevity through autophagy, with potential transgenerational benefits [41]. Taken together, these findings underscore the complex interplay between early-life and subsequent nutritional status in shaping later health.

Beyond these main results, the negative associations were more pronounced in men than women, consistent with sex-specific epigenetic patterns observed in the Dutch famine [42]. Notably, maternal and paternal estimates differed in direction: maternal exposure was associated with higher offspring BAI, whereas paternal exposure was linked to lower BAI, BRI, WHtR, COI, and ABSI. These patterns were consistent with our previous findings [10,11]. The negative intergenerational associations of paternal exposure with body indices also align with the Överkalix cohort, where prepubertal paternal famine exposure was associated with reduced diabetes and cardiovascular risk and longer lifespan in grandsons [8,34].

Our findings raise the hypothesis of a sex-specific, lineage-dependent cycle of phenotypic transmission. Several plausible mechanisms may underlie these patterns. First, epigenetic chromatin modification may transmit famine effects across generations [41]. Malnutrition during critical developmental windows can induce an irreversible “thrifty phenotype” [43], perpetuated through programmed metabolic changes [44] and epigenetic marks [42], with paternally expressed genes generally promoting fetal growth and maternally expressed genes restricting it [45]. Second, non-biological pathways, including altered household energy dynamics [46], socioeconomic trajectories [47,48], and health behaviors [49], may contribute to the clustering of obesity-related phenotypes across generations [50]. Future studies integrating molecular and detailed family and household data are needed to elucidate the underlying mechanisms.

Our prior work linked early-life famine exposure to shorter stature across two generations and sex-specific obesity risks in offspring (higher for maternal exposure, lower for paternal childhood exposure), as measured by BMI and waist circumference [9,10,11]. We observed a similar intergenerational pattern for adiposity indices, with stronger associations for BAI, BRI, and WHtR than for COI and ABSI. BAI, BRI, and WHtR are more strongly linked to abdominal obesity and metabolic disease prediction [19,51,52], whereas ABSI correlates more closely with all-cause mortality [18] and COI with mortality in older Chinese adults [53]. This suggests that offspring of famine-exposed mothers may face elevated cardio-metabolic risk in adulthood, though studies incorporating metabolic biomarkers or prospective cardiometabolic outcomes are warranted to confirm the health implications.

This study has several strengths. The CHNS is a large, longitudinal, household-based cohort covering diverse Chinese provinces, enabling examination of famine exposure at different developmental periods across two generations. We assessed sex-specific associations by stratifying F1 analysis by sex, evaluating individual and combined maternal and paternal exposure in relation to offspring indices, and testing offspring sex as a modifier. Mixed-effects models accommodated repeated measures with varying timing and completeness, maximizing use of available data.

Several limitations should be noted. First, famine exposure was defined by birth year rather than individual nutritional status, which may have misclassified those born in 1958 or 1963; however, sensitivity analyses excluding these years yielded similar results. Second, small subgroup sizes may have limited power for stratified analyses by exposure timing, though sensitivity analyses confirmed robust patterns. Third, birth cohort effects and secular trends may partly explain the observed differences, as unexposed offspring were born slightly later. We restricted the analysis to individuals born between 1955 and 1966 to mitigate this, though residual cohort effects may still have contributed to between-group differences despite famine effects generally exceeding secular trends [23]. Fourth, small subgroup sizes and multiple comparisons may have produced chance findings; however, subgroup and sensitivity analyses showed consistent patterns. Fifth, offspring analyses were restricted to biologically linked parent–offspring pairs with available parental birth information, potentially introducing selection bias and limiting generalizability and sample size. Nevertheless, as an exploratory study, these findings may inform future intergenerational research. Finally, survivor bias is possible, though a recent Dutch famine study found no difference in BMI polygenic scores between exposed and unexposed individuals [54], arguing against strong genetic or survival selection effects.

## 5. Conclusions

In summary, this study extends the DOHaD framework by demonstrating sex-specific, intergenerational associations between early-life undernutrition and body shape indices. With approximately 181 million children under 5 worldwide currently experiencing severe food poverty [55], preventing maternal and childhood malnutrition remains an urgent global health priority. Future research with longer follow-up and larger samples is warranted to validate these findings, clarify underlying mechanisms, and inform evidence-based interventions.

## Figures and Tables

**Table 1:** Characteristics ^†^ of China Health and Nutrition Survey participants (F1) by famine exposure timing in early life.

	Famine Exposure Timing			*p* ^‡^
	Unexposed	Fetal-Exposed	Childhood-Exposed	
**Men**				
Number of participants	992	679	858	
Number of observations	3530	2565	3437	
Age at survey (year)	40.5 ± 6.9	44.3 ± 6.8	48.4 ± 6.9	<0.001
High school education or above	1320 (37.4)	1348 (52.6)	1304 (37.9)	<0.001
Living in rural area	2378 (67.4)	1700 (66.3)	2249 (65.4)	0.232
Severe famine exposure	1536 (43.5)	959 (37.4)	1505 (43.8)	<0.001
Level of physical activity				<0.001
Low	1163 (32.9)	1032 (40.2)	1321 (38.4)	
Medium	832 (23.6)	574 (22.4)	699 (20.3)	
High	1535 (43.5)	959 (37.4)	1417 (41.2)	
Energy intake (kcal/d)	2480 ± 643	2466 ± 653	2467 ± 667	0.606
Smoking	2291 (64.9)	1759 (68.6)	2309 (67.2)	0.008
Alcohol drinking	2350 (66.6)	1781 (69.4)	2359 (68.6)	0.042
**Women**				
Number of participants	1067	713	847	
Number of observations	3993	2941	3625	
Age at survey (years)	40.5 ± 6.9	43.9 ± 6.9	48.2 ± 6.8	<0.001
High school education or above	1107 (27.7)	1161 (39.5)	977 (27.0)	<0.001
Living in rural area	2674 (67.0)	1996 (67.9)	2426 (66.9)	0.664
Severe famine exposure	1754 (43.9)	1075 (36.6)	1578 (43.5)	<0.001
Level of physical activity				0.007
Low	1833 (45.9)	1361 (46.3)	1746 (48.2)	
Medium	598 (15.0)	411 (14.0)	439 (12.1)	
High	1562 (39.1)	1169 (39.7)	1440 (39.7)	
Energy intake (kcal/d)	2107 ± 562	2125 ± 584	2111 ± 563	0.419
Smoking	94 (2.4)	68 (2.3)	118 (3.3)	0.020
Alcohol drinking	388 (9.7)	306 (10.4)	323 (8.9)	0.121

^†^ Data presented as mean ± standard deviation for continuous variables or *n* (%) for categorical variables. ^‡^ *p* values for the differences among subgroups using analysis of variance or Kruskal–Wallis tests (continuous variables) or chi-square tests (categorical variables).

## Data Availability

Data can be accessed via the link https://www.cpc.unc.edu/projects/china (accessed on 1 February 2025).

## Supplementary Materials

The following supporting information can be downloaded at: https://www.mdpi.com/article/10.3390/nu18152544/s1, Table S1: Characteristics of child and adult offspring by parental early-life famine exposure; Table S2: Adjusted means and 95% confident intervals of body shape indices in child offspring by parental early-life famine exposure; Table S3: Associations of parental early-life famine exposure with BAI in child offspring by sex; Table S4: Adjusted means and 95% confidence intervals of body shape indices in adult offspring by parental early-life famine exposure; Figure S1: The directed acyclic graph (DAG) for covariate selection; Figure S2: Associations of early-life famine exposure with body shape indices later in life in the F1 generation by age group; Figure S3: Associations of parental early-life famine exposure timing with body shape indices in offspring; Figure S4: Associations of parental early-life famine exposure with body shape indices in offspring by age group; Figure S5: Sensitivity analyses examining the associations of early-life famine exposure with body shape indices in later-life among F1 generation; Figure S6: Sensitivity analyses examining the associations of parental early-life famine exposure with body shape indices in child offspring; Figure S7: Sensitivity analyses examining the associations of parental early-life famine exposure with body shape index in adult offspring; Figure S8: Sensitivity analyses examining the associations of early-life famine exposure with later-life body shape indices in F1 generation using birth balanced control.

## Abbreviations

The following abbreviations are used in this manuscript:

ABSI	a body shape index
BAI	body adiposity index
BMI	body mass index
BRI	body roundness index
CHNS	the China Health and Nutrition Survey
CI	confidence interval
DOHaD	developmental origins of health and disease
LMM	linear mixed effect model
P0M0	neither parent exposed to famine
P1M0	paternal exposure only
P0M1	maternal exposure only
P1M1	both parents exposed to famine
SD	standard deviation
WHtR	waist-to-height ratio
