# Supplementary material for: Sex-Specific Associations of Famine Exposure in Early Life with Body Shape Indices Across Two Generations in China
Source: Nutrients. 2026 Aug 4;18(15):2544. doi: 10.3390/nu18152544 (PMC13468686; doi:10.3390/nu18152544)
Supplement: Supplementary file 1 [file nutrients-18-02544-s001.zip › nutrients-4440009-supplementary.pdf]

Table S1 Characteristics of child and adult offspring by parental early-life famine exposure

	Parental exposure status[1]				P values[2]
	**P0M0**	**P0M1**	**P1M0**	**P1M1**	
**Child offspring (7-17 years)**					
Number of subjects	583	113	345	1074	
Number of observations	1193	215	680	1855	
Girls (n, %)	529 (44.3)	91 (42.3)	327 (48.1)	912 (49.2)	0.029
Age at survey(year)	11.8±2.9	12.0±2.8	12.1±2.8	12.6±2.8	<0.001
Birth year (range)	1982-2006	1983-2004	1981-2003	1977-2004	<0.001
Years of education (years)	5.6±2.8	6.1±2.7	5.8±2.7	6.4±2.6	<0.001
Higher parental education (n, %)[3]	515 (43.2)	102 (47.4)	365 (53.7)	1004 (54.1)	<0.001
Rural area (n, %)	831 (69.7)	179 (83.3)	444 (65.3)	1264 (68.1)	<0.001
Severer famine (n, %)	578 (48.4)	86 (40)	422 (62.1)	831 (44.8)	<0.001
Low physical activity (n, %)	136 (11.4)	15 (7.0)	75 (11.0)	147 (7.9)	<0.001
Energy intake (kcal/d)					
Boys	1957±592	2131±570	2060±600	2089±604	<0.001
Girls	1715±511	1852±469	1824±523	1880±519	<0.001
**Adult offspring (≥18 years)**					
Number of subjects	265	53	168	669	
Number of observations	392	79	287	1222	
Women (n, %)	127 (32.4)	23 (29.1)	100 (34.8)	440 (36.0)	0.407
Age at survey (year)	22.6±3.4	23.4±3.7	22.9±3.8	23.8±4.3	<0.001
Birth year (range)	1982-1997	1983-1995	1982-1995	1974-1997	<0.001
Years of education (years)	11.2±2.8	11.4±2.4	11±2.6	10.9±3.0	0.317
Higher parental education (n, %)[3]	156 (39.8)	40 (50.6)	145 (50.5)	618 (50.6)	0.002
Rural area (n, %)	249 (63.5)	61 (77.2)	188 (65.5)	733 (60.0)	0.008
Severer famine (n, %)	156 (39.8)	27 (34.2)	177 (61.7)	529 (43.3)	<0.001
Physical activity (n, %)					0.001
Low	196 (50.0)	37 (46.8)	149 (51.9)	580 (47.5)	
Medium	142 (36.2)	34 (43.0)	111 (38.7)	409 (33.5)	
High	54 (13.8)	8 (10.1)	27 (9.4)	233 (19.1)	
Smoking (n, %)	150 (38.3)	24 (30.4)	86 (30.0)	378 (30.9)	0.041
Alcohol drinking (n, %)	156 (39.8)	31 (39.2)	98 (34.1)	440 (36.0)	0.409
Energy intake (kcal/d)					
Men	2362±500	2222±600	2318±563	2390±593	0.101
Women	1791±434	1880±441	1859±466	1869±504	0.449

Data presented as mean ± standard deviation for continuous variables, range for birth year, and n (%) for categorical variables.

[1] **P0M0**: neither parent exposed to famine; **P0M1**: maternal exposed only; **P1M0**: paternal exposed only; **P1M1**: both parents exposed to famine.

[2] P value for the difference between exposed groups using analysis of variance or Kruskal–Wallis tests (continuous variables) or chi-square tests (categorical variables).

[3] Higher parental education: at least one parent completed high school or above.

Table S2 Adjusted means and 95% confident intervals of body shape indices in child offspring by parental early-life famine exposure

	Number of observations	Adjusted mean (95%CI)				
		BAI	BRI	WHtR	COI	ABSI
**Boys**						
**Paternal exposure**						
Unexposed	772-781	24.62 (24.34, 24.90)	2.24 (2.18, 2.29)	0.4336 (0.4305, 0.4368)	1.1474 (1.1403, 1.1544)	0.1416 (0.1407, 0.1424)
Fetal exposed	651-655	24.74 (24.44, 25.04)	2.26 (2.20, 2.32)	0.4346 (0.4312, 0.4380)	1.1483 (1.1406, 1.1559)	0.1417 (0.1408, 0.1426)
Childhood exposed	627-632	24.36 (24.05, 24.67)	2.17 (2.11, 2.23)	0.4298 (0.4264, 0.4333)	1.1415 (1.1336, 1.1493)	0.1408 (0.1399, 0.1418)
**Maternal exposure**						
Unexposed	998-1009	24.80 (24.56, 25.05)	2.24 (2.19, 2.29)	0.4339 (0.4311, 0.4366)	1.1466 (1.1404, 1.1528)	0.1415 (0.1407, 0.1423)
Fetal exposed	673-679	24.38 (24.08, 24.68)*	2.20 (2.15, 2.26)	0.4313 (0.4279, 0.4346)	1.1440 (1.1364, 1.1515)	0.1411 (0.1402, 0.1421)
Childhood exposed	379-381	24.33 (23.93, 24.73)*	2.21 (2.14, 2.29)	0.4326 (0.4281, 0.4371)	1.1473 (1.1372, 1.1574)	0.1416 (0.1403, 0.1428)
**Parental exposure**						
P0M0	649-658	24.63 (24.33, 24.94)	2.24 (2.18, 2.30)	0.4339 (0.4305, 0.4373)	1.1500 (1.1423, 1.1577)	0.1419 (0.1409, 0.1428)
P0M1	121-124	24.55 (23.84, 25.25)	2.21 (2.08, 2.34)	0.4320 (0.4241, 0.4398)	1.1332 (1.1156, 1.1508)	0.1400 (0.1378, 0.1421)
P1M0	349-351	25.12 (24.71, 25.53)	2.24 (2.16, 2.32)	0.4338 (0.4291, 0.4384)	1.1402 (1.1298, 1.1507)	0.1408 (0.1395, 0.1421)
P1M1	929-936	24.34 (24.08, 24.59)	2.21 (2.16, 2.26)	0.4317 (0.4289, 0.4346)	1.1467 (1.1403, 1.1532)	0.1415 (0.1407, 0.1422)
**Girls**						
**Paternal exposure**						
Unexposed	613-617	25.59 (25.26, 25.91)	2.11 (2.06, 2.17)	0.4262 (0.4229, 0.4295)	1.1271 (1.1194, 1.1347)	0.1391 (0.1382, 0.1400)
Fetal exposed	574-578	25.66 (25.33, 26.00)	2.14 (2.09, 2.20)	0.4283 (0.4248, 0.4317)	1.1297 (1.1218, 1.1376)	0.1394 (0.1385, 0.1404)
Childhood exposed	647-652	25.66 (25.35, 25.98)	2.12 (2.06, 2.17)	0.4265 (0.4232, 0.4298)	1.1250 (1.1175, 1.1325)	0.1389 (0.1379, 0.1398)
**Maternal exposure**						
Unexposed	845-853	25.56 (25.29, 25.84)	2.11 (2.06, 2.16)	0.4258 (0.4230, 0.4287)	1.1254 (1.1189, 1.1320)	0.1389 (0.1381, 0.1397)
Fetal exposed	587-593	25.70 (25.37, 26.02)	2.14 (2.09, 2.20)	0.4278 (0.4244, 0.4312)	1.1309 (1.1231, 1.1387)	0.1395 (0.1386, 0.1405)
Childhood exposed	400-403	25.71 (25.31, 26.12)	2.14 (2.07, 2.21)	0.4280 (0.4239, 0.4322)	1.1253 (1.1158, 1.1349)	0.1390 (0.1378, 0.1401)
**Parental exposure**						
P0M0	523-528	25.51 (25.16, 25.86)	2.10 (2.04, 2.16)	0.4252 (0.4216, 0.4288)	1.1253 (1.1170, 1.1336)	0.1389 (0.1379, 0.1399)
P0M1	89-90	26.03 (25.19, 26.88)	2.21 (2.06, 2.35)	0.4322 (0.4234, 0.4409)	1.1369 (1.1168, 1.1570)	0.1404 (0.1379, 0.1428)
P1M0	322-325	25.65 (25.20, 26.09)	2.12 (2.05, 2.20)	0.4270 (0.4224, 0.4315)	1.1255 (1.1150, 1.1361)	0.1390 (0.1377, 0.1402)
P1M1	898-905	25.67 (25.40, 25.94)	2.13 (2.09, 2.18)	0.4275 (0.4247, 0.4302)	1.1278 (1.1215, 1.1342)	0.1392 (0.1384, 0.1400)

Abbreviations: **BAI**, body adiposity index; **BRI**, body roundness index; **WHtR**, waist to height ratio; **COI**, conicity index; **ABSI**, a body shape index (for adolescent); **CI**, confidence interval; **P0M0**, neither parent exposed to famine; **P1M0**, paternal exposure only; **P0M1**, maternal exposure only; **P1M1**, both parents exposed to famine.

Number of Observations: available observations for each body shape index of the eligible participants.

Mean (95%CI) estimated using linear regression models adjusting for age at survey and sex. * P<0.05 compared with the unexposed group.

The values of WHtR, COI, and ABSI are presented with four decimal places to reflect the small differences between groups.

Table S3 Associations of parental early-life famine exposure with BAI in child offspring by sex

	Boys		Girls		P for interaction
	**No. of Obs**	**β (95%CI)**	**No. of Obs**	**β (95%CI)**	
**Paternal exposure**					*0.091*
Unexposed	779	Ref	613	Ref	
Fetal exposed	655	0.36 (-0.14, 0.87)	577	0.22 (-0.35, 0.79)	
Childhood exposed	631	0.21 (-0.44, 0.86)	648	0.03 (-0.67, 0.73)	
**Maternal exposure**					*0.007*
Unexposed	1009	Ref	845	Ref	
Fetal exposed	675	**-0.50 (-1.00, 0.00)**	590	0.32 (-0.24, 0.87)	
Childhood exposed	381	-0.52 (-1.23, 0.19)	403	0.05 (-0.70, 0.80)	
**Paternal exposure**					*0.017*
P0M0	658	Ref	523	Ref	
P0M1	121	0.07 (-0.78, 0.92)	90	0.53 (-0.49, 1.55)	
P1M0	351	0.60 (0.03, 1.18)	322	0.27 (-0.37, 0.91)	
P1M1	935	-0.21 (-0.66, 0.23)	903	0.30 (-0.20, 0.80)	

Abbreviations: **BAI**, body adiposity index; **CI**, confidence interval. **No. of Obs**, available observations for each body shape index of the eligible participants. **P0M0**, neither parent exposed to famine; **P1M0**, paternal exposure only; **P0M1**, maternal exposure only; **P1M1**, both parents exposed to famine.

No. of Obs: number of available observations for each body shape index of the eligible participants.

The β with 95%CI represents the difference in each body shape index between the exposed and unexposed groups, estimated using linear mixed effect models with fractional polynomials for age and adjusted for sex, residential areas (urban or rural), educational level (high or low), and severity of famine (less or more).

P for interaction between paternal, maternal, and parental famine exposure and sex.

Table S4 Adjusted means and 95% confidence intervals of body shape indices in adult offspring by parental early-life famine exposure

	Number of observations	Adjusted mean (95%CI)				
		BAI	BRI	WHtR	COI	ABSI
**Male**						
**Paternal exposure**						
Unexposed	270-318	23.83 (23.40, 24.25)	2.89 (2.78, 2.99)	0.4710 (0.4651, 0.4768)	1.1983 (1.1877, 1.2090)	0.0779 (0.0772, 0.0786)
Fetal exposed	352-399	23.77 (23.40, 24.14)	2.97 (2.87, 3.06)	0.4748 (0.4696, 0.4800)	1.2009 (1.1913, 1.2104)	0.0780 (0.0773, 0.0786)
Childhood exposed	531-566	23.36 (23.06, 23.66)	2.70 (2.62, 2.78)*	0.4604 (0.4560, 0.4648)*	1.1807 (1.1727, 1.1887)*	0.0771 (0.0766, 0.0777)
**Maternal exposure**						
Unexposed	392-449	23.59 (23.24, 23.95)	2.85 (2.76, 2.94)	0.4689 (0.4640, 0.4739)	1.1966 (1.1875, 1.2056)	0.0779 (0.0773, 0.0785)
Fetal exposed	372-426	23.70 (23.34, 24.06)	2.90 (2.81, 2.99)	0.4713 (0.4663, 0.4764)	1.1933 (1.1840, 1.2025)	0.0775 (0.0769, 0.0781)
Childhood exposed	389-408	23.49 (23.14, 23.85)	2.73 (2.63, 2.82)	0.4619 (0.4567, 0.4671)	1.1835 (1.1740, 1.1929)	0.0773 (0.0767, 0.0779)
**Parental exposure**						
P0M0	222-263	23.53 (23.06, 23.99)	2.84 (2.73, 2.96)	0.4687 (0.4622, 0.4751)	1.1993 (1.1875, 1.2111)	0.0781 (0.0773, 0.0789)
P0M1	48-55	25.20 (24.20, 26.19)*	3.09 (2.84, 3.35)	0.4816 (0.4676, 0.4957)	1.1933 (1.1677, 1.2188)	0.0770 (0.0753, 0.0787)
P1M0	170-186	23.68 (23.15, 24.21)	2.87 (2.73, 3.01)	0.4692 (0.4615, 0.4769)	1.1926 (1.1786, 1.2066)	0.0776 (0.0767, 0.0785)
P1M1	713-779	23.49 (23.23, 23.75)	2.80 (2.73, 2.87)	0.4657 (0.4619, 0.4695)	1.1882 (1.1813, 1.1950)*	0.0774 (0.0770, 0.0779)
**Female**						
**Paternal exposure**						
Unexposed	136-149	26.52 (25.85, 27.19)	2.73 (2.57, 2.88)	0.4608 (0.4523, 0.4694)	1.1696 (1.1531, 1.1861)	0.0770 (0.0759, 0.0781)
Fetal exposed	198-213	26.61 (26.05, 27.16)	2.67 (2.55, 2.80)	0.4583 (0.4512, 0.4654)	1.1567 (1.1431, 1.1703)	0.0760 (0.0751, 0.0769)
Childhood exposed	313-324	26.19 (25.75, 26.63)	2.44 (2.34, 2.54)*	0.4455 (0.4397, 0.4513)*	1.1394 (1.1284, 1.1505)*	0.0752 (0.0744, 0.0759)*
**Maternal exposure**						
Unexposed	211-226	26.30 (25.76, 26.84)	2.60 (2.48, 2.73)	0.4543 (0.4473, 0.4613)	1.1551 (1.1417, 1.1684)	0.0761 (0.0752, 0.0770)
Fetal exposed	193-213	26.20 (25.64, 26.76)	2.57 (2.44, 2.69)	0.4520 (0.4448, 0.4592)	1.1479 (1.1342, 1.1617)	0.0756 (0.0747, 0.0765)
Childhood exposed	243-247	26.61 (26.11, 27.11)	2.55 (2.43, 2.67)	0.4521 (0.4455, 0.4588)	1.1507 (1.1380, 1.1633)	0.0757 (0.0749, 0.0766)
**Parental exposure**						
P0M0	117-126	26.40 (25.68, 27.13)	2.69 (2.53, 2.86)	0.4593 (0.4499, 0.4687)	1.1671 (1.1491, 1.1850)	0.0769 (0.0757, 0.0781)
P0M1	19-23	27.18 (25.39, 28.96)	2.89 (2.49, 3.28)	0.4687 (0.4469, 0.4904)	1.1831 (1.1409, 1.2253)	0.0775 (0.0747, 0.0803)
P1M0	94-100	26.17 (25.37, 26.98)	2.49 (2.30, 2.68)	0.4481 (0.4376, 0.4585)	1.1403 (1.1204, 1.1603)	0.0752 (0.0739, 0.0765)
P1M1	417-437	26.39 (26.01, 26.78)	2.54 (2.45, 2.63)	0.4512 (0.4462, 0.4562)	1.1476 (1.1381, 1.1572)	0.0756 (0.0749, 0.0762)

Abbreviations: **BAI**, body adiposity index; **BRI**, body roundness index; **WHtR**, waist to height ratio; **COI**, conicity index; **ABSI**, a body shape index; **CI**, confidence interval; **P0M0**, neither parent exposed to famine; **P1M0**, paternal exposure only; **P0M1**, maternal exposure only; **P1M1**, both parents exposed to famine.

No. of Obs: number of available observations for each body shape index of the eligible participants.

Mean (95%CI) estimated using linear regression models adjusting for age at survey and sex. * $P<0.05$ compared with the unexposed group.

The values of WHtR, COI, and ABSI are presented with four decimal places to reflect the small differences between groups.

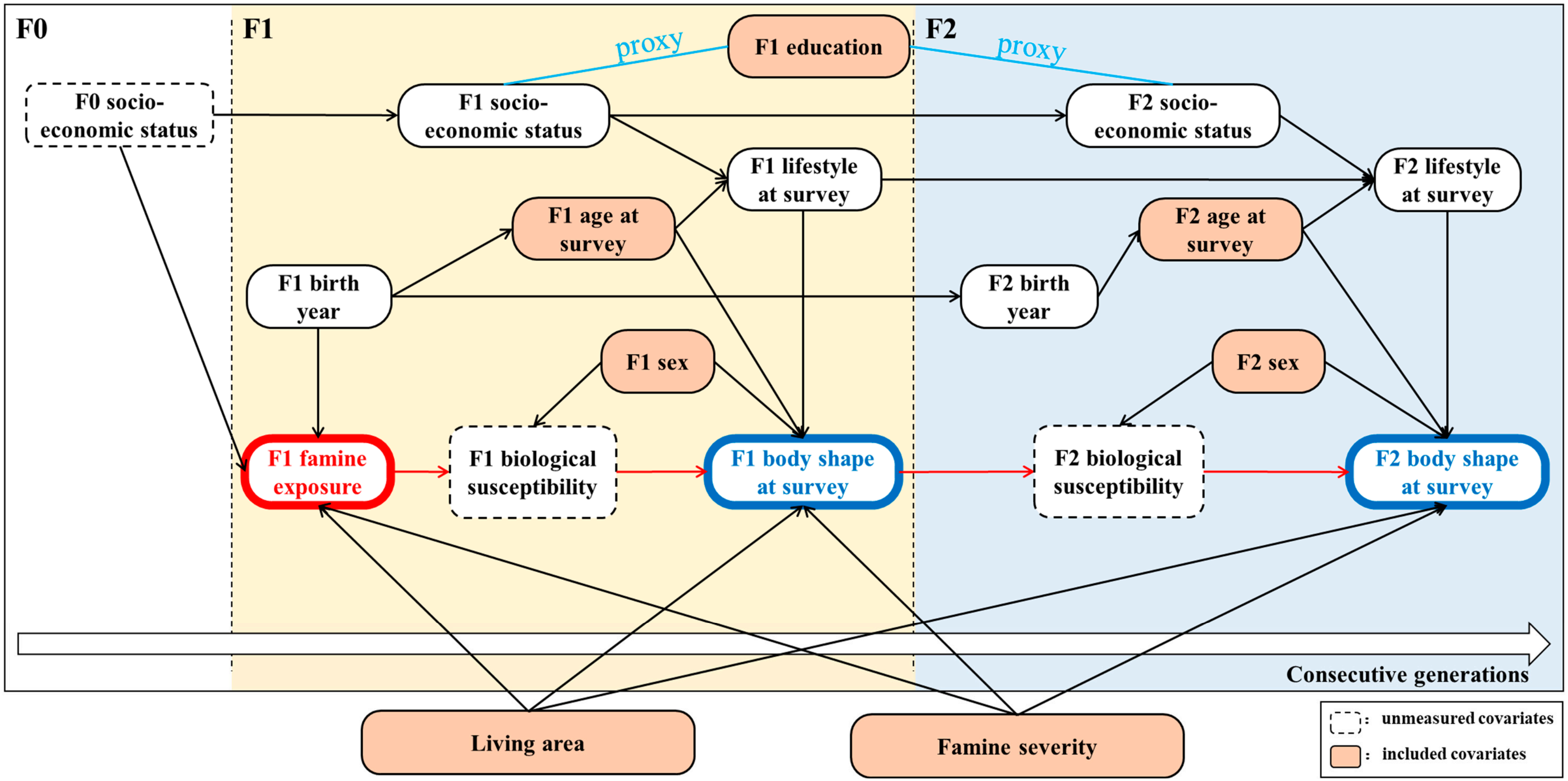

Figure S1 The directed acyclic graph (DAG) for covariate selection

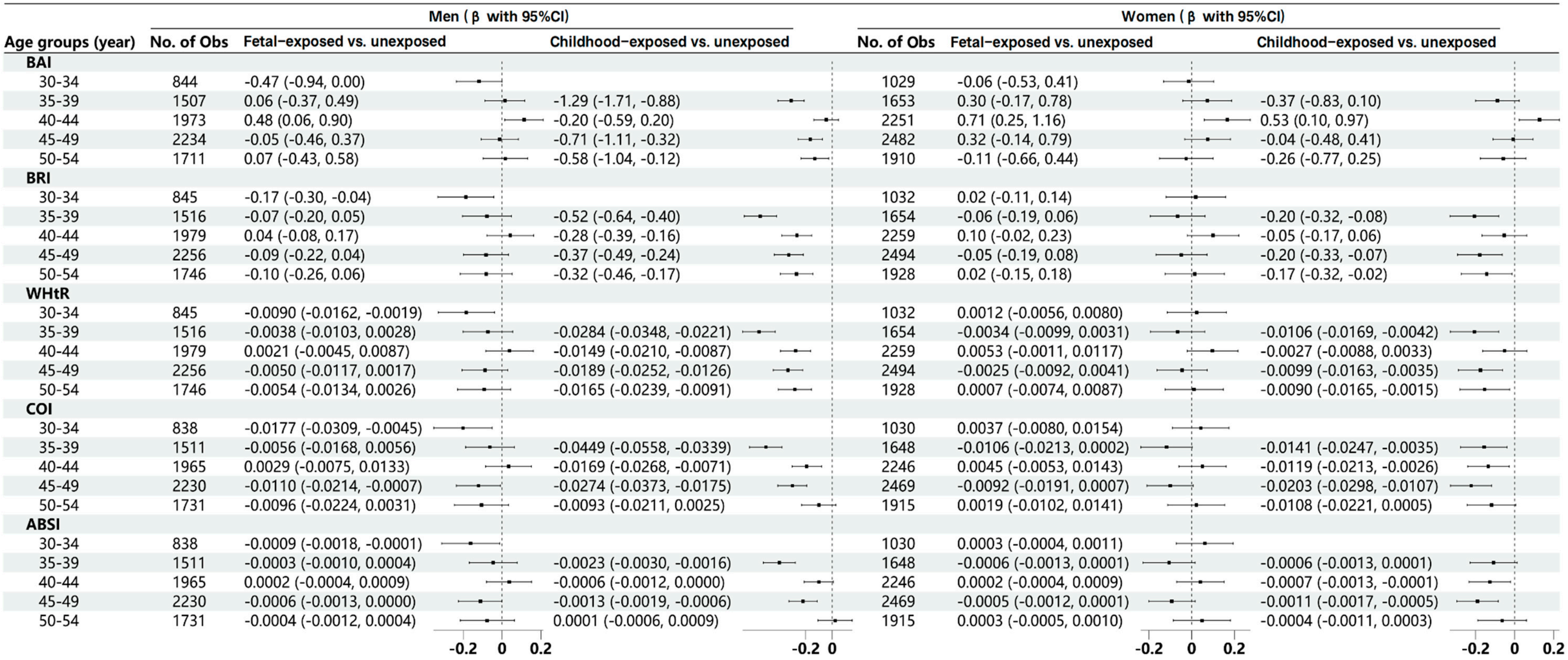

Age groups (year)	Men: No. of Obs	Men: Fetal–exposed vs. unexposed	Men: Childhood–exposed vs. unexposed	Women: No. of Obs	Women: Fetal–exposed vs. unexposed	Women: Childhood–exposed vs. unexposed
**BAI**						
30-34	844	-0.47 (-0.94, 0.00)		1029	-0.06 (-0.53, 0.41)	
35-39	1507	0.06 (-0.37, 0.49)	-1.29 (-1.71, -0.88)	1653	0.30 (-0.17, 0.78)	-0.37 (-0.83, 0.10)
40-44	1973	0.48 (0.06, 0.90)	-0.20 (-0.59, 0.20)	2251	0.71 (0.25, 1.16)	0.53 (0.10, 0.97)
45-49	2234	-0.05 (-0.46, 0.37)	-0.71 (-1.11, -0.32)	2482	0.32 (-0.14, 0.79)	-0.04 (-0.48, 0.41)
50-54	1711	0.07 (-0.43, 0.58)	-0.58 (-1.04, -0.12)	1910	-0.11 (-0.66, 0.44)	-0.26 (-0.77, 0.25)
**BRI**						
30-34	845	-0.17 (-0.30, -0.04)		1032	0.02 (-0.11, 0.14)	
35-39	1516	-0.07 (-0.20, 0.05)	-0.52 (-0.64, -0.40)	1654	-0.06 (-0.19, 0.06)	-0.20 (-0.32, -0.08)
40-44	1979	0.04 (-0.08, 0.17)	-0.28 (-0.39, -0.16)	2259	0.10 (-0.02, 0.23)	-0.05 (-0.17, 0.06)
45-49	2256	-0.09 (-0.22, 0.04)	-0.37 (-0.49, -0.24)	2494	-0.05 (-0.19, 0.08)	-0.20 (-0.33, -0.07)
50-54	1746	-0.10 (-0.26, 0.06)	-0.32 (-0.46, -0.17)	1928	0.02 (-0.15, 0.18)	-0.17 (-0.32, -0.02)
**WHtR**						
30-34	845	-0.0090 (-0.0162, -0.0019)		1032	0.0012 (-0.0056, 0.0080)	
35-39	1516	-0.0038 (-0.0103, 0.0028)	-0.0284 (-0.0348, -0.0221)	1654	-0.0034 (-0.0099, 0.0031)	-0.0106 (-0.0169, -0.0042)
40-44	1979	0.0021 (-0.0045, 0.0087)	-0.0149 (-0.0210, -0.0087)	2259	0.0053 (-0.0011, 0.0117)	-0.0027 (-0.0088, 0.0033)
45-49	2256	-0.0050 (-0.0117, 0.0017)	-0.0189 (-0.0252, -0.0126)	2494	-0.0025 (-0.0092, 0.0041)	-0.0099 (-0.0163, -0.0035)
50-54	1746	-0.0054 (-0.0134, 0.0026)	-0.0165 (-0.0239, -0.0091)	1928	0.0007 (-0.0074, 0.0087)	-0.0090 (-0.0165, -0.0015)
**COI**						
30-34	838	-0.0177 (-0.0309, -0.0045)		1030	0.0037 (-0.0080, 0.0154)	
35-39	1511	-0.0056 (-0.0168, 0.0056)	-0.0449 (-0.0558, -0.0339)	1648	-0.0106 (-0.0213, 0.0002)	-0.0141 (-0.0247, -0.0035)
40-44	1965	0.0029 (-0.0075, 0.0133)	-0.0169 (-0.0268, -0.0071)	2246	0.0045 (-0.0053, 0.0143)	-0.0119 (-0.0213, -0.0026)
45-49	2230	-0.0110 (-0.0214, -0.0007)	-0.0274 (-0.0373, -0.0175)	2469	-0.0092 (-0.0191, 0.0007)	-0.0203 (-0.0298, -0.0107)
50-54	1731	-0.0096 (-0.0224, 0.0031)	-0.0093 (-0.0211, 0.0025)	1915	0.0019 (-0.0102, 0.0141)	-0.0108 (-0.0221, 0.0005)
**ABSI**						
30-34	838	-0.0009 (-0.0018, -0.0001)		1030	0.0003 (-0.0004, 0.0011)	
35-39	1511	-0.0003 (-0.0010, 0.0004)	-0.0023 (-0.0030, -0.0016)	1648	-0.0006 (-0.0013, 0.0001)	-0.0006 (-0.0013, 0.0001)
40-44	1965	0.0002 (-0.0004, 0.0009)	-0.0006 (-0.0012, 0.0000)	2246	0.0002 (-0.0004, 0.0009)	-0.0007 (-0.0013, -0.0001)
45-49	2230	-0.0006 (-0.0013, 0.0000)	-0.0013 (-0.0019, -0.0006)	2469	-0.0005 (-0.0012, 0.0001)	-0.0011 (-0.0017, -0.0005)
50-54	1731	-0.0004 (-0.0012, 0.0004)	0.0001 (-0.0006, 0.0009)	1915	0.0003 (-0.0005, 0.0010)	-0.0004 (-0.0011, 0.0003)

Figure S2 Associations of early-life famine exposure with body shape indices later in life in the F1 generation by age group

Abbreviations: **BAI**, body adiposity index; **BRI**, body roundness index; **WHtR**, waist to height ratio; **COI**, conicity index; **ABSI**, a body shape index; **CI**: confidence interval;

No. of obs, number of available observations for each body shape index of the eligible participants.

The β with 95%CI represents the difference in each body shape index between the exposed and unexposed groups, estimated using linear mixed effect models with fractional polynomials for age and adjusted for sex, residential areas (urban or rural), educational level (high or low), and severity of famine (less or more).

All p values <0.05 for the interactions between famine exposure and age groups for five indices.

The values of WHtR, COI, and ABSI are presented with four decimal places to reflect the small differences between groups.

Results presented in the forest plots derived from the standardized values (z-scores) for each index.

	Among child offspring		Among adult offspring	
	No. of Obs	β (95%CI)	No. of Obs	β (95%CI)
**BAI**				
**Parental exposure timing**				
p0m0	1181	Ref	339	Ref
p0m1	211	0.25 (-0.41, 0.91)	67	1.20 (0.08, 2.32)
p1m0	550	0.46 (0.00, 0.92)	216	0.04 (-0.71, 0.78)
p1m1	600	0.05 (-0.40, 0.50)	270	0.18 (-0.52, 0.88)
p1m2	82	0.37 (-0.62, 1.37)	64	1.76 (0.53, 2.99)
p2m0	123	0.16 (-0.68, 1.01)	48	-0.41 (-1.80, 0.98)
p2m1	454	0.13 (-0.36, 0.61)	228	-0.24 (-0.98, 0.50)
p2m2	702	-0.06 (-0.48, 0.36)	568	-0.12 (-0.72, 0.47)
**BRI**				
**Parental exposure timing**				
p0m0	1185	Ref	389	Ref
p0m1	213	0.03 (-0.09, 0.16)	78	0.17 (-0.09, 0.44)
p1m0	552	0.04 (-0.04, 0.13)	233	0.04 (-0.14, 0.22)
p1m1	599	0.01 (-0.08, 0.09)	309	0.03 (-0.14, 0.19)
p1m2	82	0.11 (-0.08, 0.29)	70	0.38 (0.08, 0.67)
p2m0	124	-0.12 (-0.28, 0.03)	53	-0.40 (-0.73, -0.06)
p2m1	460	-0.02 (-0.11, 0.07)	252	-0.11 (-0.29, 0.07)
p2m2	700	-0.02 (-0.09, 0.06)	585	-0.17 (-0.32, -0.03)
**WHtR**				
**Parental exposure timing**				
p0m0	1185	Ref	389	Ref
p0m1	213	0.0022 (-0.0050, 0.0095)	78	0.0087 (-0.0059, 0.0233)
p1m0	552	0.0029 (-0.0022, 0.0080)	233	0.0015 (-0.0086, 0.0116)
p1m1	599	0.0003 (-0.0047, 0.0053)	309	0.0010 (-0.0082, 0.0103)
p1m2	82	0.0065 (-0.0044, 0.0175)	70	0.0207 (0.0044, 0.0370)
p2m0	124	-0.0072 (-0.0166, 0.0021)	53	-0.0219 (-0.0404, -0.0033)
p2m1	460	-0.0013 (-0.0067, 0.0040)	252	-0.0064 (-0.0163, 0.0034)
p2m2	700	-0.0004 (-0.0050, 0.0042)	585	-0.0096 (-0.0177, -0.0016)
**COI**				
**Parental exposure timing**				
p0m0	1174	Ref	384	Ref
p0m1	212	-0.0051 (-0.0204, 0.0101)	77	0.0025 (-0.0232, 0.0282)
p1m0	548	-0.0020 (-0.0127, 0.0087)	230	-0.0025 (-0.0200, 0.0150)
p1m1	596	0.0037 (-0.0068, 0.0143)	303	-0.0041 (-0.0202, 0.0120)
p1m2	81	-0.0056 (-0.0291, 0.0179)	70	0.0191 (-0.0087, 0.0469)
p2m0	124	-0.0209 (-0.0404, -0.0014)	53	-0.0306 (-0.0620, 0.0007)
p2m1	452	-0.0064 (-0.0177, 0.0050)	250	-0.0198 (-0.0369, -0.0027)
p2m2	698	-0.0024 (-0.0123, 0.0074)	582	-0.0205 (-0.0344, -0.0066)
**ABSI**				
**Parental exposure timing**				
p0m0	1174	Ref	384	Ref
p0m1	212	-0.0005 (-0.0024, 0.0014)	77	-0.0004 (-0.0020, 0.0013)
p1m0	548	-0.0001 (-0.0014, 0.0012)	230	-0.0003 (-0.0015, 0.0008)
p1m1	596	0.0004 (-0.0009, 0.0017)	303	-0.0004 (-0.0014, 0.0007)
p1m2	81	-0.0004 (-0.0032, 0.0025)	70	0.0005 (-0.0013, 0.0023)
p2m0	124	-0.0025 (-0.0049, -0.0002)	53	-0.0015 (-0.0035, 0.0006)
p2m1	452	-0.0008 (-0.0022, 0.0006)	250	-0.0013 (-0.0024, -0.0002)
p2m2	698	-0.0003 (-0.0015, 0.0009)	582	-0.0011 (-0.0021, -0.0002)

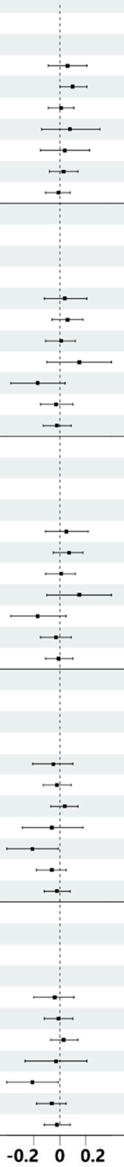

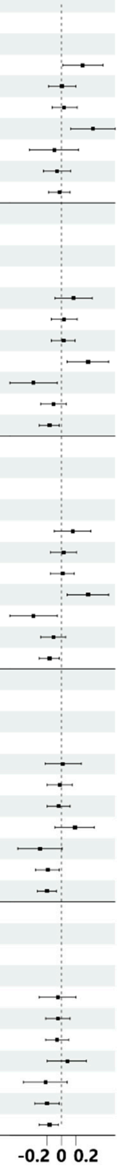

Figure S3 Associations of parental early-life famine exposure timing with body shape indices in offspring

Abbreviations: **BAI**, body adiposity index; **BRI**, body roundness index; **WHtR**, waist to height ratio; **COI**, conicity index; **ABSI**, a body shape index; **CI**, confidence interval; **p0m0**, neither parent exposed to famine; **p0m1**: unexposed father and fetal-exposed mother; **p1m0**: fetal-exposed father and unexposed mother; **p1m1**: both parents fetal-exposed to famine; **p1m2**: fetal-exposed father and childhood-exposed mother; **p2m0**: childhood-exposed father and unexposed mother; **p2m1**: childhood-exposed father and fetal-exposed mother; **p2m2**: both parents childhood-exposed to famine.

No. of Obs: number of available observations for each body shape index of the eligible participants.
The β with 95%CI represents the difference in each body shape index between the exposed and unexposed groups, estimated using linear mixed effect models with fractional polynomials for age and adjusted for sex, residential areas (urban or rural), educational level (high or low), and severity of famine (less or more).
The values of WHtR, COI, and ABSI are presented with four decimal places to reflect the small differences between groups.
Results presented in the forest plots derived from the standardized values (z-scores) for each index.

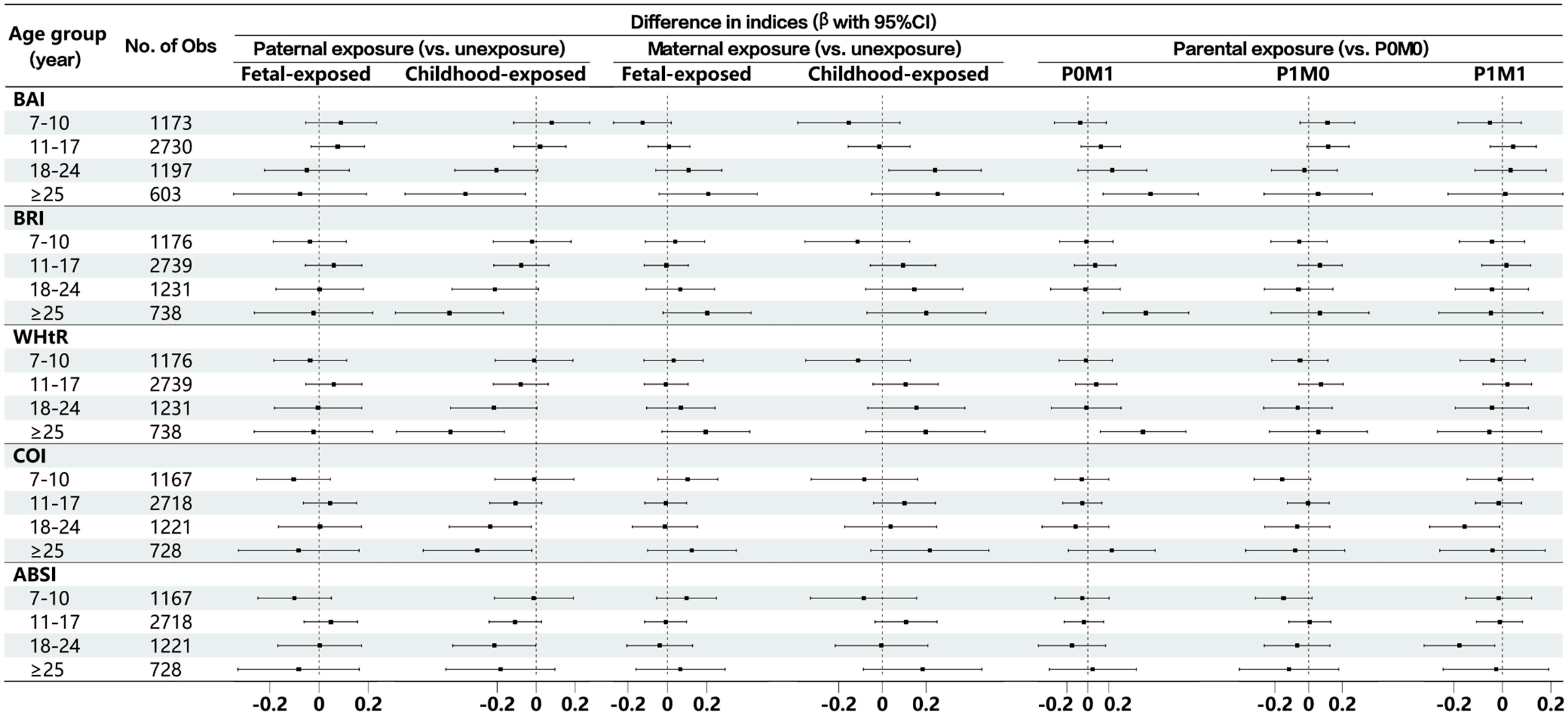

Figure S4 Associations of parental early-life famine exposure with body shape indices in offspring by age group

Abbreviations: **BAI**, body adiposity index; **BRI**, body roundness index; **WHtR**, waist to height ratio; **COI**, conicity index; **ABSI**, a body shape index; **CI**, confidence interval; **No. of Obs**, available observations for each body shape index of the eligible participants; **P1M0**: paternal exposure only; **P0M1**: maternal exposure only; **P1M1**: both parents exposed to famine.

The β with 95%CI represents the difference in each body shape index between the exposed and unexposed groups, estimated using linear mixed effect models with fractional polynomials for age and adjusted for sex, residential areas (urban or rural), educational level (high or low), and severity of famine (less or more).

The values of WHtR, COI, and ABSI presented with four decimal places to reflect the small differences between groups. All p values <0.05 for the interactions between parental famine exposure and age groups for five indices. Results presented in the forest plots derived from the standardized values (z-scores) for each index.

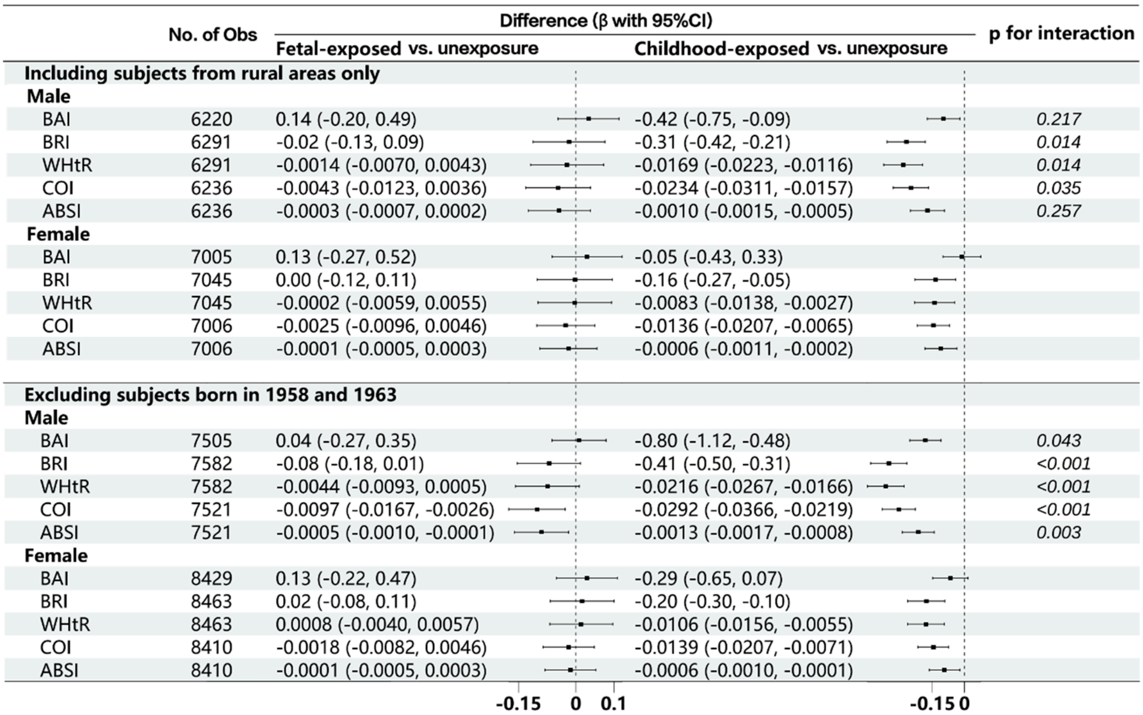

	No. of Obs	Difference (β with 95%CI) Fetal-exposed vs. unexposure	Difference (β with 95%CI) Childhood-exposed vs. unexposure	p for interaction
**Including subjects from rural areas only**				
**Male**				
BAI	6220	0.14 (-0.20, 0.49)	-0.42 (-0.75, -0.09)	*0.217*
BRI	6291	-0.02 (-0.13, 0.09)	-0.31 (-0.42, -0.21)	*0.014*
WHtR	6291	-0.0014 (-0.0070, 0.0043)	-0.0169 (-0.0223, -0.0116)	*0.014*
COI	6236	-0.0043 (-0.0123, 0.0036)	-0.0234 (-0.0311, -0.0157)	*0.035*
ABSI	6236	-0.0003 (-0.0007, 0.0002)	-0.0010 (-0.0015, -0.0005)	*0.257*
**Female**				
BAI	7005	0.13 (-0.27, 0.52)	-0.05 (-0.43, 0.33)	
BRI	7045	0.00 (-0.12, 0.11)	-0.16 (-0.27, -0.05)	
WHtR	7045	-0.0002 (-0.0059, 0.0055)	-0.0083 (-0.0138, -0.0027)	
COI	7006	-0.0025 (-0.0096, 0.0046)	-0.0136 (-0.0207, -0.0065)	
ABSI	7006	-0.0001 (-0.0005, 0.0003)	-0.0006 (-0.0011, -0.0002)	
**Excluding subjects born in 1958 and 1963**				
**Male**				
BAI	7505	0.04 (-0.27, 0.35)	-0.80 (-1.12, -0.48)	*0.043*
BRI	7582	-0.08 (-0.18, 0.01)	-0.41 (-0.50, -0.31)	*<0.001*
WHtR	7582	-0.0044 (-0.0093, 0.0005)	-0.0216 (-0.0267, -0.0166)	*<0.001*
COI	7521	-0.0097 (-0.0167, -0.0026)	-0.0292 (-0.0366, -0.0219)	*<0.001*
ABSI	7521	-0.0005 (-0.0010, -0.0001)	-0.0013 (-0.0017, -0.0008)	*0.003*
**Female**				
BAI	8429	0.13 (-0.22, 0.47)	-0.29 (-0.65, 0.07)	
BRI	8463	0.02 (-0.08, 0.11)	-0.20 (-0.30, -0.10)	
WHtR	8463	0.0008 (-0.0040, 0.0057)	-0.0106 (-0.0156, -0.0055)	
COI	8410	-0.0018 (-0.0082, 0.0046)	-0.0139 (-0.0207, -0.0071)	
ABSI	8410	-0.0001 (-0.0005, 0.0003)	-0.0006 (-0.0010, -0.0001)	

Figure S5 Sensitivity analyses examining the associations of early-life famine exposure with body shape indices in later-life among F1 generation

Abbreviations: **BAI**, body adiposity index; **BRI**, body roundness index; **WHtR**, waist to height ratio; **COI**, conicity index; **ABSI**, a body shape index; **ABSI_a**, ABSI for adolescent; **CI**, confidence interval.
No. of Obs, available observations for each body shape index of the eligible participants.
Mean (95%CI) estimated using linear regression models adjusting for age at body measurement and sex (among all subjects only).
The β with 95%CI represents the difference in each body shape index between the exposed and unexposed groups, estimated using linear mixed effect models with fractional polynomials for age and adjusted for sex, residential areas (urban or rural), educational level (high or low), and severity of famine (less or more).
p for interaction between famine exposure with sex.
The values of WHtR, COI, and ABSI are presented with four decimal places to reflect the small differences between groups.
Results presented in the forest plots derived from the standardized values (z-scores) for each index.

	No. of Obs	BAI	BRI	WHtR	COI	ABSI
		Difference (β with 95%CI)				
**Including subjects from rural areas only**						
**Paternal exposure**						
Unexposed	998-1001	Ref	Ref	Ref	Ref	Ref
Fetal-exposed	814-817	-0.06 (-0.51, 0.39)	-0.04 (-0.12, 0.04)	-0.0023 (-0.0072, 0.0026)	-0.0084 (-0.0187, 0.0019)	-0.0010 (-0.0023, 0.0002)
Childhood-exposed	874-881	-0.27 (-0.83, 0.29)	-0.11 (-0.22, -0.01)	-0.0069 (-0.0130, -0.0008)	-0.0137 (-0.0266, -0.0008)	-0.0017 (-0.0033, -0.0002)
**Maternal exposure**						
Unexposed	1264-1270	Ref	Ref	Ref	Ref	Ref
Fetal-exposed	883-889	-0.02 (-0.46, 0.42)	0.00 (-0.08, 0.08)	0.0000 (-0.0048, 0.0048)	-0.0015 (-0.0116, 0.0087)	-0.0002 (-0.0014, 0.0011)
Childhood-exposed	542-543	0.04 (-0.57, 0.66)	0.08 (-0.03, 0.20)	0.0055 (-0.0012, 0.0122)	0.0061 (-0.0081, 0.0203)	0.0009 (-0.0009, 0.0026)
**Parental exposure**						
P0M0	822-827	Ref	Ref	Ref	Ref	Ref
P0M1	176-177	0.24 (-0.48, 0.97)	0.02 (-0.12, 0.15)	0.0015 (-0.0065, 0.0095)	-0.0119 (-0.0286, 0.0048)	-0.0013 (-0.0033, 0.0008)
P1M0	440-443	0.04 (-0.48, 0.56)	-0.04 (-0.14, 0.05)	-0.0024 (-0.0081, 0.0033)	-0.0148 (-0.0267, -0.0028)	-0.0017 (-0.0032, -0.0003)
P1M1	1249-1255	-0.17 (-0.57, 0.22)	-0.05 (-0.12, 0.02)	-0.0028 (-0.0072, 0.0016)	-0.0107 (-0.0199, -0.0015)	-0.0013 (-0.0024, -0.0002)
**Excluding parents born in 1958 and 1963**						
**Paternal exposure**						
Unexposed	755-764	Ref	Ref	Ref	Ref	Ref
Fetal-exposed	940-947	0.46 (-0.06, 0.97)	0.01 (-0.09, 0.11)	0.0007 (-0.0052, 0.0065)	-0.0029 (-0.0155, 0.0098)	-0.0003 (-0.0018, 0.0013)
Childhood-exposed	753-763	0.21 (-0.47, 0.89)	-0.08 (-0.21, 0.05)	-0.0046 (-0.0123, 0.0031)	-0.0123 (-0.0291, 0.0045)	-0.0015 (-0.0036, 0.0005)
**Maternal exposure**						
Unexposed	1025-1031	Ref	Ref	Ref	Ref	Ref
Fetal-exposed	976-988	-0.29 (-0.80, 0.22)	0.03 (-0.07, 0.13)	0.0016 (-0.0042, 0.0073)	0.0063 (-0.0063, 0.0189)	0.0007 (-0.0008, 0.0023)
Childhood-exposed	451-454	-0.01 (-0.73, 0.72)	0.07 (-0.07, 0.20)	0.0045 (-0.0037, 0.0126)	0.0110 (-0.0068, 0.0289)	0.0014 (-0.0008, 0.0036)
**Parental exposure**						
P0M0	686-691	Ref	Ref	Ref	Ref	Ref
P0M1	69-73	-0.44 (-1.52, 0.64)	0.08 (-0.12, 0.28)	0.0042 (-0.0078, 0.0163)	-0.0091 (-0.0353, 0.0172)	-0.0008 (-0.0041, 0.0024)
P1M0	338-340	0.40 (-0.18, 0.98)	0.02 (-0.09, 0.13)	0.0011 (-0.0055, 0.0077)	-0.0080 (-0.0223, 0.0062)	-0.0008 (-0.0026, 0.0009)
P1M1	1355-1367	0.12 (-0.29, 0.53)	0.01 (-0.07, 0.09)	0.0004 (-0.0043, 0.0051)	-0.0010 (-0.0112, 0.0091)	-0.0001 (-0.0013, 0.0011)
**Using birth-balanced control group**						
**Paternal exposure**						
Birth-balanced control	2660-2682	Ref	Ref	Ref	Ref	Ref
Fetal-exposed	1225-1233	0.23 (-0.09, 0.54)	0.04 (-0.02, 0.10)	0.0026 (-0.0009, 0.0061)	0.0036 (-0.0037, 0.0110)	0.0005 (-0.0004, 0.0014)
**Maternal exposure**						
Birth-balanced control	2625-2643	Ref	Ref	Ref	Ref	Ref
Fetal-exposed	1260-1272	-0.04 (-0.35, 0.27)	-0.01 (-0.07, 0.05)	-0.0010 (-0.0044, 0.0025)	-0.0001 (-0.0074, 0.0071)	-0.0001 (-0.0009, 0.0008)
**Parental exposure**						
P0M0	1996-2009	Ref	Ref	Ref	Ref	Ref
P0M1	664-673	0.18 (-0.21, 0.57)	0.01 (-0.06, 0.08)	0.0004 (-0.0039, 0.0047)	-0.0038 (-0.0130, 0.0053)	-0.0004 (-0.0016, 0.0007)
P1M0	629-634	0.46 (0.05, 0.86)	0.07 (-0.01, 0.14)	0.0040 (-0.0005, 0.0085)	-0.0003 (-0.0097, 0.0091)	0.0001 (-0.0011, 0.0012)
P1M1	596-600	0.06 (-0.36, 0.48)	0.02 (-0.06, 0.10)	0.0008 (-0.0038, 0.0055)	0.0058 (-0.0040, 0.0156)	0.0007 (-0.0005, 0.0018)
		-0.3 0	0 0.3	-0.3 0 0.3	-0.3 0	-0.3 0

Figure S6 Sensitivity analyses examining the associations of parental early-life famine exposure with body shape indices in child offspring

Abbreviations: **BAI**, body adiposity index; **BRI**, body roundness index; **WHtR**, waist to height ratio; **COI**, conicity index; **ABSI**, a body shape index (for adolescent); **CI**, confidence interval; **No. of obs**, available observations for each body shape index of the eligible participants; **P0M0**, neither parent exposed to famine; **P1M0**, paternal exposure only; **P0M1**, maternal exposure only; **P1M1**, both parents exposed to famine.

The β with 95%CI represents the difference in each body shape index between the exposed and unexposed groups, estimated using linear mixed effect models with fractional polynomials for age and adjusted for sex, residential areas (urban or rural), educational level (high or low), and severity of famine (less or more).

Birth-balanced control: combined unexposed group and childhood-exposed group as a control group.

The values of WHtR, COI, and ABSI are presented with four decimal places to reflect the small differences between groups.

Results presented in the forest plots derived from the standardized values (z-scores) for each index.

	No. of Obs	Difference (β with 95%CI) BAI	BRI	WHtR	COI	ABSI
**Including subjects from rural areas only**						
**Paternal exposure**						
Unexposed	256-307	Ref	Ref	Ref	Ref	Ref
Fetal-exposed	346-397	-0.54 (-1.28, 0.20)	-0.07 (-0.25, 0.10)	-0.0040 (-0.0136, 0.0056)	-0.0060 (-0.0229, 0.0110)	-0.0003 (-0.0014, 0.0008)
Childhood-exposed	483-518	-1.50 (-2.39, -0.61)	-0.41 (-0.62, -0.20)	-0.0223 (-0.0338, -0.0108)	-0.0317 (-0.0518, -0.0116)	-0.0015 (-0.0028, -0.0002)
**Maternal exposure**						
Unexposed	374-434	Ref	Ref	Ref	Ref	Ref
Fetal-exposed	351-409	0.49 (-0.21, 1.20)	0.12 (-0.05, 0.28)	0.0060 (-0.0032, 0.0152)	0.0042 (-0.0119, 0.0203)	0.0000 (-0.0011, 0.0010)
Childhood-exposed	360-379	1.33 (0.43, 2.22)	0.26 (0.05, 0.47)	0.0141 (0.0025, 0.0257)	0.0121 (-0.0079, 0.0321)	0.0003 (-0.0010, 0.0016)
**Parental exposure**						
P0M0	205-247	Ref	Ref	Ref	Ref	Ref
P0M1	51-60	1.44 (0.18, 2.70)	0.24 (-0.06, 0.53)	0.0116 (-0.0048, 0.0280)	0.0061 (-0.0231, 0.0353)	-0.0002 (-0.0021, 0.0017)
P1M0	169-187	-0.25 (-1.12, 0.62)	-0.08 (-0.28, 0.13)	-0.0045 (-0.0161, 0.0070)	-0.0101 (-0.0304, 0.0101)	-0.0007 (-0.0020, 0.0007)
P1M1	660-728	-0.14 (-0.79, 0.51)	-0.09 (-0.24, 0.06)	-0.0050 (-0.0135, 0.0034)	-0.0143 (-0.0294, 0.0007)	-0.0010 (-0.0019, 0.0000)
**Excluding parents born in 1958 and 1963**						
**Paternal exposure**						
Unexposed	184-219	Ref	Ref	Ref	Ref	Ref
Fetal-exposed	423-476	-0.09 (-0.98, 0.80)	-0.05 (-0.25, 0.16)	-0.0027 (-0.0140, 0.0087)	-0.0076 (-0.0277, 0.0126)	-0.0004 (-0.0017, 0.0009)
Childhood-exposed	539-570	-1.12 (-2.25, 0.01)	-0.34 (-0.60, -0.07)	-0.0186 (-0.0331, -0.0041)	-0.0309 (-0.0564, -0.0054)	-0.0015 (-0.0032, 0.0001)
**Maternal exposure**						
Unexposed	288-329	Ref	Ref	Ref	Ref	Ref
Fetal-exposed	423-486	0.30 (-0.53, 1.13)	0.03 (-0.16, 0.23)	0.0011 (-0.0096, 0.0119)	-0.0059 (-0.0249, 0.0131)	-0.0006 (-0.0018, 0.0007)
Childhood-exposed	435-450	0.72 (-0.36, 1.80)	0.04 (-0.22, 0.29)	0.0022 (-0.0119, 0.0162)	-0.0044 (-0.0290, 0.0201)	-0.0005 (-0.0021, 0.0011)
**Parental exposure**						
P0M0	164-192	Ref	Ref	Ref	Ref	Ref
P0M1	20-27	2.06 (0.03, 4.08)	0.36 (-0.08, 0.80)	0.0170 (-0.0074, 0.0414)	0.0097 (-0.0331, 0.0525)	0.0000 (-0.0028, 0.0028)
P1M0	124-137	0.28 (-0.72, 1.27)	0.03 (-0.20, 0.26)	0.0010 (-0.0119, 0.0140)	-0.0040 (-0.0272, 0.0192)	-0.0003 (-0.0018, 0.0012)
P1M1	838-909	-0.11 (-0.83, 0.61)	-0.16 (-0.33, 0.01)	-0.0094 (-0.0187, -0.0002)	-0.0258 (-0.0423, -0.0093)	-0.0016 (-0.0027, -0.0005)
**Using birth-balanced control group**						
**Paternal exposure**						
Birth-balanced control	1250-1357	Ref	Ref	Ref	Ref	Ref
Fetal-exposed	550-612	0.32 (-0.15, 0.79)	0.16 (0.04, 0.27)	0.0084 (0.0021, 0.0147)	0.0130 (0.0022, 0.0239)	0.0006 (-0.0001, 0.0013)
**Maternal exposure**						
Birth-balanced control	1235-1330	Ref	Ref	Ref	Ref	Ref
Fetal-exposed	565-639	0.04 (-0.43, 0.50)	0.01 (-0.10, 0.12)	0.0003 (-0.0059, 0.0065)	-0.0036 (-0.0143, 0.0071)	-0.0003 (-0.0010, 0.0004)
**Parental exposure**						
P0M0	955-1027	Ref	Ref	Ref	Ref	Ref
P0M1	295-330	0.20 (-0.38, 0.79)	0.07 (-0.07, 0.21)	0.0036 (-0.0043, 0.0114)	-0.0016 (-0.0152, 0.0119)	-0.0004 (-0.0013, 0.0005)
P1M0	280-303	0.49 (-0.11, 1.09)	0.22 (0.08, 0.37)	0.0119 (0.0037, 0.0201)	0.0152 (0.0011, 0.0292)	0.0006 (-0.0003, 0.0015)
P1M1	270-309	0.26 (-0.36, 0.87)	0.13 (-0.01, 0.28)	0.0069 (-0.0011, 0.0150)	0.0083 (-0.0058, 0.0223)	0.0003 (-0.0006, 0.0012)
		-0.3 0 0.3	-0.3 0 0.3	-0.3 0 0.3	-0.3 0 0.3	-0.3 0 0.3

Figure S7 Sensitivity analyses examining the associations of parental early-life famine exposure with body shape index in adult offspring

Abbreviations: **BAI**, body adiposity index; **BRI**, body roundness index; **WHtR**, waist to height ratio; **COI**, conicity index; **ABSI**, a body shape index; **CI**, confidence interval; **No. of obs**, available observations for each body shape index of the eligible participants; **P0M0**, neither parent exposed to famine; **P1M0**, paternal exposure only; **P0M1**, maternal exposure only; **P1M1**, both parents exposed to famine.

The β with 95%CI represents the difference in each body shape index between the exposed and unexposed groups, estimated using linear mixed effect models with fractional polynomials for age and adjusted for sex, residential areas (urban or rural), educational level (high or low), and severity of famine (less or more).

Birth-balanced control: combined unexposed group and childhood-exposed group as a control group.

The values of WHtR, COI, and ABSI are presented with four decimal places to reflect the small differences between groups.

Results presented in the forest plots derived from the standardized values (z-scores) for each index.

	Means with 95%CI				Difference (β with 95%CI)
	**No. of Obs**	**Control**	**No. of Obs**	**Fetal-exposed**	**Fetal-exposed vs. control**
**Male**					
BAI	6853	25.49 (25.41, 25.57)	2521	25.89 (25.76, 26.02)	0.37 (0.12, 0.62)
BRI	6926	3.36 (3.34, 3.39)	2545	3.50 (3.46, 3.54)	0.11 (0.03, 0.19)
WHtR	6926	0.4970 (0.4957, 0.4983)	2545	0.5043 (0.5022, 0.5063)	0.0057 (0.0016, 0.0097)
COI	6867	1.2221 (1.2200, 1.2242)	2531	1.2284 (1.2249, 1.2319)	0.0044 (-0.0013, 0.0101)
ABSI	6867	0.0789 (0.0788, 0.0791)	2531	0.0791 (0.0788, 0.0793)	0.0001 (-0.0002, 0.0004)
**Female**					
BAI	7521	29.96 (29.87, 30.05)	2910	30.15 (30.01, 30.30)	0.30 (0.01, 0.59)
BRI	7567	3.57 (3.55, 3.60)	2915	3.62 (3.58, 3.66)	0.10 (0.02, 0.18)
WHtR	7567	0.5075 (0.5063, 0.5088)	2915	0.5100 (0.5079, 0.5120)	0.0052 (0.0012, 0.0093)
COI	7520	1.2032 (1.2012, 1.2052)	2896	1.2047 (1.2015, 1.2080)	0.0045 (-0.0008, 0.0097)
ABSI	7520	0.0776 (0.0775, 0.0777)	2896	0.0776 (0.0774, 0.0778)	0.0002 (-0.0001, 0.0005)

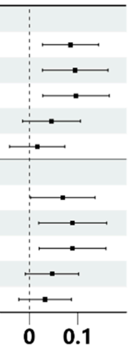

Figure S8 Sensitivity analyses examining the associations of early-life famine exposure with later-life body shape indices in F1 generation using birth balanced control

Abbreviations: **BAI**, body adiposity index; **BRI**, body roundness index; **WHtR**, waist to height ratio; **COI**, conicity index; **ABSI**, a body shape index; **CI**, confidence interval.
No. of Obs: number of available observations for each body shape index of the eligible participants.
Mean (95%CI) estimated using linear regression models adjusting for age at survey and sex.
Control: combined unexposed group and childhood-exposed group as a control group.
The β with 95%CI represents the difference in each body shape index between the exposed and unexposed groups, estimated using linear mixed effect models with fractional polynomials for age and adjusted for sex, residential areas (urban or rural), educational level (high or low), and severity of famine (less or more).
The values of WHtR, COI, and ABSI are presented with four decimal places to reflect the small differences between groups.
Results presented in the forest plots derived from the standardized values (z-scores) for each index.
